# Differential MR/GR Activation in Mice Results in Emotional States Beneficial or Impairing for Cognition

**DOI:** 10.1155/2007/90163

**Published:** 2007-04-15

**Authors:** Vera Brinks, Maaike H. van der Mark, E. Ron de Kloet, Melly S. Oitzl

**Affiliations:** Gorlaeus Lab, Division of Medical Pharmacology, LACDR/LUMC, Leiden University, Einsteinweg 55, 2300 Leiden, The Netherlands

## Abstract

Corticosteroids regulate stress response and influence emotion, learning, and memory via two receptors in the brain, the high-affinity mineralocorticoid (MR) and low-affinity glucocorticoid receptor (GR). We test the hypothesis that MR- and GR-mediated effects interact in emotion and cognition when a novel situation is encountered that is relevant for a learning process. By adrenalectomy and additional constant corticosterone supplement we obtained four groups of male C57BL/6J mice with differential chronic MR and GR activations. Using a hole board task, we found that mice with continuous predominant MR and moderate GR activations were fast learners that displayed low anxiety and arousal together with high directed explorative behavior. Progressive corticosterone concentrations with predominant action via GR induced strong emotional arousal at the expense of cognitive performance. These findings underline the importance of a balanced MR/GR system for emotional and cognitive functioning that is critical for mental health.

## 1. INTRODUCTION

Stress and emotions facilitate or impair learning and memory processes [1]. Glucocorticoids are the stress hormones secreted from the adrenals after activation of the hypothal-amus-pituitary-adrenal (HPA) axis; that is, corticosterone in rats and mice, cortisol in humans. The effect on synaptic plasticity and memory formation is mediated by two types of nuclear receptors: MR (mineralocorticoid receptor) and GR (glucocorticoid receptor) which are located in areas involved in emotion, learning, and memory. While MR is present in the hippocampus and to lesser extent in the prefrontal cortex, amygdale, and paraventricular nucleus [2–5], GR can be found throughout the brain with high levels in the hippocampus and paraventricular nucleus [5]. Other characteristics are the differential affinities for corticosterone: MR has a tenfold higher affinity than GR, resulting in predominant MR occupation during low basal levels and additional GR activation during increased corticosterone concentration due to stress or circadian peak activity of the hypothalamic-pituitary-adrenal (HPA) axis [6]. The precise involvement of MR and GR in emotion and cognition is still debated.

Animal studies have shown that activation or blockade of either receptor influences behavior related to anxiety, exploration, and memory. These behaviors are linked to the limbic system and are part of the behavioral repertoire tested in spatial memory tasks and also in fear conditioning [7]. With respect to unconditioned fear-related behavior, Smythe et al. [8] have described that MR modulates anxiety-like behavior of rats in the light/dark box. Oitzl et al. have shown that intracerebroventricular injection of a rather selective MR antagonist in rats influenced corticosterone-induced behavioral reactivity to spatial novelty [9]. Recent findings in mutant mice with inactivated MR in the forebrain (Cre-loxP recombination [10]) support the pharmacologically detected role of MR on the modulation of behavioral strategies. Loss of the limbic MR impaired behavioral plasticity, evidenced by a differential performance during the first exposure to learning tasks, that is, their behavioral reactivity to novelty. In contrast, learning slopes in the water and radial arm maze were not affected. This increased behavioral reactivity to novel objects was observed in the face of normal anxiety-like behavior in the open field and elevated-O-maze [10]. Indeed, it should be clarified whether MR affects anxiety or appropriate context-dependent behavioral reactivity.

Others suggest that adaptive behavior is modulated by a combined MR/GR mediated action. An example is the inhibition of corticosterone production and thus prevention of GR activation in the face of full MR activation: this led to decreased fear-induced immobility and fear-related anxiety in rats [11]. Complementary, exogenous corticosterone application or prior social defeat increased anxiogenic behavior in rats tested in the elevated plus maze 24 hours later. Antagonism of the GR in the lateral septum eliminated the anxiogenic effect [12]. Interesting in this study is the 24-hour delay, indicating involvement of memory. Indeed, GR is implicated in memory consolidation processes, demonstrated by using GR-agonists and GR-antagonists in rats, chickens, as well as GR mutant mice [13–18]. Calvo and Volosin have shown that corticosterone-induced effects on anxiety after restraint stress require both MR and GR [19]. Taken together, MR appears to be responsible for the immediate facilitative effects of corticosterone on memory acquisition, while the modulation of spatial and fear memory relies on the presence of a functional GR [20]. To disentangle the combined contribution of MR and GR to most adequate performance, we will study the functions of these receptors in a task that allows simultaneous registration of emotional and memory parameters.

How emotion and cognition affect each other is still relatively unknown. Forgas and George suggested that a stimulus first needs to be identified before the appropriate emotional response will follow [21]. Others focus more on the neurobiological process of emotion and cognition, which can be functionally, anatomically, and even pharmacologically separated [22]. We hypothesize that emotion and cognition are interdependent and both will be affected by differential MR and GR activations: we propose that the two corticosteroid receptors MR and GR contribute differentially but in a coordinated way to information processing.

The aim of this study was to examine how MR and GR interact in information processing presented by emotional and learning/memory elements of a task. Next to the well-known use of MR and GR antagonists, MR/GR activation ratios can be endocrinologically and pharmacologically adjusted by removal of the adrenals (adrenalectomy (ADX)) and additional subcutaneous corticosterone pellet implantation. In contrast to rats, mice that undergo adrenalectomy remain to produce low concentrations of corticosterone from scattered cell groups in the vicinity of the adrenals [23–25]. Therefore, ADXed mice provide an excellent model for predominant MR activation. Different degrees of continuous GR activation can be achieved via corticosterone released from implanted pellets. We used this approach and tested mice in the modified hole board [26] measuring behaviors that define general activity, emotions, motivation, and learning and memory. Subsequent principal component analysis will allow to determine the correlation between emotions and cognition.

## 2. MATERIAL AND METHODS

### 2.1. Animals

Forty eight 12-week-old male C57BL/6 mice were obtained from Charles River (Maastricht, The Netherlands). After arrival, the mice were housed individually in the experimental room with sawdust bedding, water and food *ad libitum*, at 20°C with controlled humidity under a 12 h : 12 h light/dark cycle (lights on at 08.00 am) for at least one week. To familiarize with the bait used in the modified hole board task, all mice received a few pieces of almonds daily in the week before surgery. All experiments were approved by the committee on Animal Health and Care from the Leiden University, The Netherlands, and were performed in strict compliance with the EEC recommendations for the care and use of laboratory animals.

### 2.2. Endocrine manipulation of MR/GR activation

Mice were randomly selected for one of the following groups and operated accordingly: (i) sham-operated (Sham), (ii) adrenalectomized mice (ADX), (iii) adrenalectomized mice with an additional low corticosterone pellet (ALC), or (iv) adrenalectomized mice with an additional high corticosterone pellet (AHC).

#### 2.2.1. Surgery

Mice were gas anaesthetized with a mixture of isoflurane/nitrous oxide (4% isoflurane bolus followed by 2% isoflurane). Body temperature was kept constant at 37°C by a heating pad. Adrenals were removed (ADX) using the dorsal approach followed by subcutaneous pellet implantation on the flank of the animal. While in rats ADX removes the endogenous source of corticosterone, in mice it clamps corticosterone to low concentrations comparable to the circadian trough of adrenally intact mice. Accessory adrenocortical cells secrete stable amounts of corticosterone [23–25, 27] that maintain extensive occupation of MR. Stress or circadian rhythm does not lead to a rise in corticosterone in ADX mice. High circulating levels of ACTH indicate the lack of GR activation; that is, no negative feedback.

Sham operation involved the same procedures as adrenalectomy except for the removal of the adrenals. Surgery was performed between 10.00 and 12.00 am and lasted maximally 10 minutes per mouse. Adrenals were removed within 5 minutes. After surgery, all mice received an additional bottle containing 0.9% salt solution. Behavioral testing started 3 days after surgery. To confirm effectiveness of the adrenalectomy and pellet implantation, plasma corticosterone levels were measured 2 days after surgery, on day 0 of the experiment, and one day after the last behavioral test on day 11. Mice with abnormal corticosterone concentrations in the blood were excluded from further analysis. This resulted in seven mice per group.

#### 2.2.2. Pellet preparation

Two types of pellets were made for subcutaneous implantation: (i) a 5% corticosterone (ICN Biomedicals, Inc., Calif, USA) 95% cholesterol pellet for moderate MR/GR activation and (ii) a 20% corticosterone 80% cholesterol pellet for strong MR/GR activation. All pellets weighed 100 mg, with a diameter of 7 mm and thickness of 2 mm and were homemade. Corticosterone dose was chosen following a pilot experiment in which plasma corticosterone concentrations of about 100 and 150 ng/mL for the 5% and 20% pellets, respectively, were measured two days after implantation.

### 2.3. Modified hole board testing

#### 2.3.1. Setup

The modified hole board consisted of an opaque grey PVC box (50 × 50 × 50 cm) with a center board (37 × 20 cm) on which 10 grey cylinders (4 cm height) were staggered in two lines [26]. Always the same three cylinders were baited with a small piece of almond on top of a grid, and were marked with a white ring. Seven other cylinders contained a nonobtainable almond underneath the grid and were marked with a black ring. The mice were placed in the modified hole board for 3 trials per day with changing start positions. One trial lasted maximally 5 minutes, or until the mouse had found the three baits. All testings were performed between 9.00–12.00 am.

#### 2.3.2. Behavioral observation

The behavior of the mice was observed, recorded, and analyzed with a semiautomatic scoring system (The Observer Mobile 4.1, Noldus Information Technology, Wageningen, The Netherlands). All measured behavioral parameters are represented in Table 1. As indication for (i) working memory, the number of repeated holevisits was calculated and (ii) reference memory, the number of visits to nonbaited holes was taken. In addition, a camera was installed above the setup to measure distance moved and velocity of the mice with an automatic tracking system (Ethovision 1.95, Noldus Information Technology, Wageningen, The Netherlands).

### 2.4. General experimental procedure

Mice were tested in the modified hole board over 10 days. On days 1 to 5 and 8, the three baited cylinders were marked with a white ring as visual cue while the remaining cylinders were marked with a black ring. This allowed visuospatial discrimination. On days 6 and 7, mice were not tested. On days 9 and 10, all rings were removed from the cylinders, but the bait remained in the same cylinders. This allowed to estimate if the mice used a spatial strategy or visual discrimination to solve the task.

A trial lasted maximally 5 minutes and was ended when the mouse had eaten all three baits.

On days 0 and 11, blood was collected via a tail incision or after decapitation. Blood plasma was used to measure corticosterone concentrations (ICN Biomedicals, Inc., Calif, USA). Because exposure to high concentrations of corticosterone results in shrinkage of the thymus, thymus weight was estimated as well.

### 2.5. Statistical analysis

Differences in corticosterone concentrations between groups and days were analyzed by two-way ANOVA (SPSS 11.5.0) with Tukey's post-hoc analysis. To analyze thymus and body-weight differences, a one-way ANOVA was performed.

The behavioral data are presented as mean of 3 trials per day ± SEM. Data were subjected to general linear model (GLM-) repeated measures with Tukey as post-hoc test to analyze progression over days and group differences per day. Furthermore, factor analysis (principal component analysis (PCA)) was performed over groups and days to obtain a more comprehensive analysis of emotional and cognitive parameters. This analysis uses cross-mouse comparisons to distinguish the relation between behavioral parameters. It includes as much data as possible in each factor to minimize residual variance from the original dataset. The PCA was performed with a varimax rotation on variables with communalities over 0.7, that is, of which 70% of the variance is explained by the factors extracted. The number of extracted factors was not predefined; factors with an eigenvalue > 1 were accepted. Factor scores were subjected to a two-way ANOVA to determine differences between groups and days. *P* < .05 was accepted as level of significance.

## 3. RESULTS

### 3.1. Behavior

#### 3.1.1. Emotion and exploration

**Figure 1:**
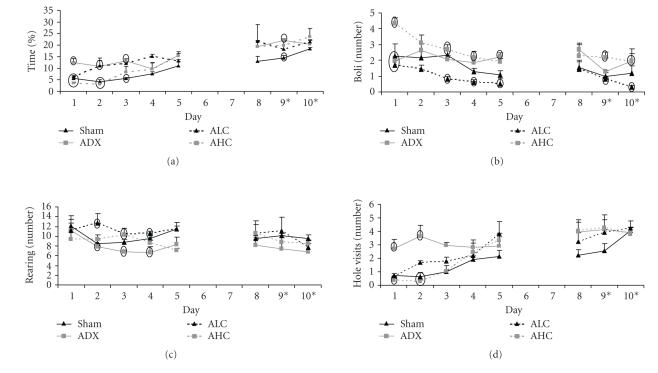
Behavior of mice in the modified hole board. (a) Percentage of time spent on center board, (b) number of defecations, (c) number of rearings, (D) number of hole visits, including revisits of sham (black line), ADX (grey line), ALC (striped black line), and AHC mice (striped grey line). Days 9 and 10 on the *x*-axis indicate removal of rings from all cylinders, while the bait remained in the same cylinders as before. Data present the mean of the three trials per day ± SEM. Ovals mark data points with significant differences *P* < .05 between groups within days.

Figure 1 shows the results for some of the emotional and explorative parameters during all days of testing in the modified hole board. Figure 1(a) illustrates that ADX followed by ALC mice have a high percentage of time spent on the center board, indicative of low anxiety [26, 28–30] during the first few days. In contrast, AHC and sham mice spent little time on the center board during this period. From day 4 on, few significant differences were found between groups. GLM from day 1 to 10 revealed a significant group/day interaction F(21,588) 2.355, *P* = .001.

Figure 1(b) shows that AHC mice display twofold more defecation compared to other groups, indicating high arousal. With repeated testing, ALC mice display less defecation compared to ADX and AHC mice. GLM revealed a significant progressive decrease over days F(21,588) 7.629, *P* < .0001, just passing statistical significance between groups (F(21,588) 1.524, *P* = .063).

The number of rearings was taken as measure for general exploration (Figure 1(c)). Comparing the first and the last days of testing, no differences were found between groups while on days 2, 3, and 4 ADX mice displayed the lowest number of rearings. GLM showed a significant change over days (F(21,588) 11.439, *P* < .0001) although not significant between groups (F(21,588) 1.25, * P* = .203).

ADX mice display highly directed exploration/behavioral reactivity on all days of testing, reaching statistical significance on days 1 and 2 as indicated by the number of hole visits (Figure 1(d)). Sham, AHC, and ALC mice start off with few hole visits which increase over time. GLM supported this by significant group/day interaction F(21,588) 1.983, *P* = .006.

Total distance moved and velocity were comparable between groups over all days of testing (data not shown).

#### 3.1.2. Cognition

**Figure 2:**
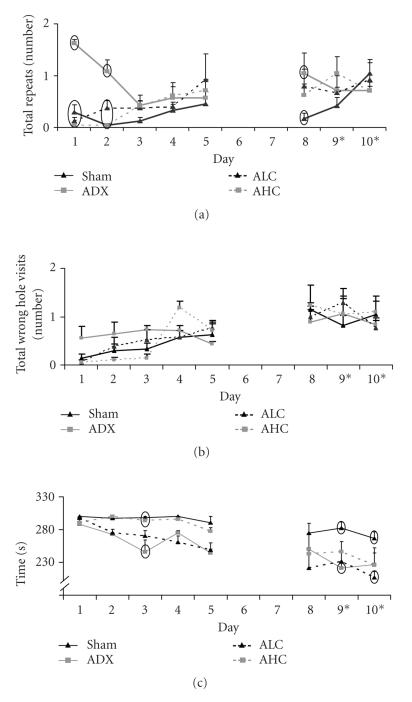
(a) Working memory expressed as number of holes revisited. (b) Reference memory expressed as visits to nonbaited holes. (c) Time to finish the task, that is, to obtain all three baits or 5 minutes, of sham (black line), ADX (grey line), ALC (striped black line), and AHC mice (striped grey line). Days 9 and 10 on the *x*-axis indicate removal of rings from all cylinders, while the bait remained in the same cylinders as before. Data present the mean of the three trials per day ± SEM. Ovals mark data points with significant differences *P* < .05 between groups within days.

Figure 2 shows the results for three cognitive parameters on all days of testing in the modified hole board. Figure 2(a) illustrates increased repeated hole visits (working memory) in ADX mice on day 8 of testing compared to sham mice. We consider the low repeated hole visits on days 1 and 2 of sham, ALC, and AHC mice as not reliable, because the total number of hole visits is also very low on these days. Over time, sham, ALC, and AHC mice show increased repeats in parallel with increased total hole visits. GLM showed a significant group/day interaction (F(21,532) 2.029, *P* = .005).

Figure 2(b) shows no significant differences in nonbaited hole visits (reference memory) between sham, ADX, ALC, and AHC mice during all days of testing.

The time to finish the task is an additional learning parameter (Figure 2(c)). ADX and ALC mice were fast learners compared to sham and AHC mice. Removal of the rings on days 9 and 10 did not influence the time to finish the task, indicating the use of a spatial learning strategy at that time of training. At the last day of testing, performance of sham mice was still poor although progression over days proved to be significant (F(21,532) 18.327, *P* = .000).

#### 3.1.3. Factor analysis

Principal component analysis (PCA) over all behavioral data resulted in the extraction of four factors (Table 2) which explain 81% of total variance. Factor 1 (41%) combines behavioral parameters that can be classified as anxiety, motivation, and good learning, Factor 2 (19%) represents directed exploration, behavioral reactivity, and working memory, Factor 3 (11%) represents general activity and Factor 4 (10%) includes behavioral parameters that can be classified as impaired learning.

One-way ANOVA between groups on factor loadings for Factor 1 (anxiety, motivation, good learning) revealed significant differences between sham mice compared to ADX, ALC, and AHC mice (F(3,279) 11.562, *P* = .000). Significant group differences were also found between ADX mice compared to sham, ALC, and AHC mice for Factor 3 (general activity; F(3,279) 8.362, *P* = .000).

Furthermore, when comparing the factor loadings over days, significant differences were found for Factor 1 between days 3 and 4 compared to days 9 and 10, (F(7,279) 4.460, *P* = .000). This indicates low anxiety, more motivation, and better learning at the end of testing in all groups. Factor 3 was significantly different between day 2 and days 1, 8, and 9 (F(7,279) 2.522, *P* = .016), which indicates that general activity was decreased at the end of testing.

### 3.2. Corticosterone and thymus weight

Plasma corticosterone and thymus weights are presented in Table 3. Both low and high corticosterone pellet groups, ALC and AHC, had higher plasma corticosterone concentrations on day 0 (F(3,31) 29.540, *P* = .0001) than the sham and ADX mice. On day 11 of the experiment, only AHC mice showed significantly increased corticosterone levels (F(3,31) 28.977, *P* = .0001), compared to sham, ADX, and ALC mice. Plasma corticosterone in sham and ADX mice remained at the same low basal morning level throughout the experiment, while corticosterone concentrations of ALC and AHC mice decreased in the course of the study (F(1,15) 7.835, *P* = .014 and F(1,15) 13.344, *P* = .003).

Thymus weights on day 11 supported the exposure to elevated corticosterone during the experiment with significantly lower thymus weights for ALC and AHC mice compared to sham and ADX mice (F(3,31) 22.332, *P* = .000). In fact, ADX mice had an enlarged thymus. ALC mice had a less shrunken thymus than AHC mice, indicating exposure to lower corticosterone concentrations than AHC. Body weight on day 11 was comparable between groups F(24,27) 1.731, *P* = .187.

## 4. DISCUSSION

Four groups of mice were generated by endocrine manipulation, resulting in different amounts of circulating corticosterone concentrations in the blood. Given the different affinities of the receptors for the hormone, we expect a differential MR/GR activation in these groups: (i) sham mice with an intact HPA axis, (ii) ADX mice with residual stable low corticosterone levels and thus continuous MR activation, (iii) ALC mice with moderate elevated circulating corticosterone concentrations allowing extensive MR and moderate GR activations, and (iv) AHC mice with a full MR and a substantial GR activation due to high circulating levels of corticosterone. We found emotional expressions and cognitive performance related to differential corticosteroid receptor activation. Continuous predominant MR activation directed emotional components indicative for less anxiety to the benefit of cognition, while continuous additional GR activation was associated with impaired learning.

### 4.1. Continuous predominant MR activation results in emotions that can be beneficial for learning

Mice with stable predominant MR activation (ADX) show increased directed exploration/behavioral reactivity towards the cylinders (hole visits) and low anxiety during the first days of testing, that is, when the setting is novel. This corresponds to the observation that transgenic mice with low GR, and rats with ICV injection of GR antagonist express low-anxiety-related behavior [31, 32]. However, it contrasts previous findings that GR blockade by single infusion of RU38486 into the hippocampus has no anxiolytic effect in rats in the light/dark box [33]. Of course, the methods to achieve predominant MR activation differ in the history of inactivated GR, species, stressed state of the animals, and behavioral task. Also a differentiation between context-related behavioral reactivity and anxiety is not possible. However, the design of the present study allows to make this distinction. Factor analysis reveals that the variables time on center board (anxiety, motivation, good learning; Factor 1) and hole visits (directed exploration and behavioral reactivity; Factor 2) are not correlated. Thus, the general idea that mice which are more prone to go to the unprotected center area are likely to display more cylinder directed behavior is not supported. In contrast, anxiety is correlated with motivation (latency to first hole visit, latency eat bait): mice with a low anxiety approach the unprotected area faster.

Overall, low anxiety and high directed exploration/behavioral reactivity could be beneficial for the onset of learning, especially during the first days of testing. We observed an apparent fast onset of learning in these predominantly MR mice. High directed exploration towards the cylinders will eventually result in finding all baits, without any necessary learning of the task. Indeed, mice of this group show an increase in working memory errors (revisits) after the two-day break without testing. GR is expected to promote the consolidation of MR-related adaptive behavior, leaving the lack of GR activation as the most likely explanation for the memory deficit. The results of the Berger study [34] can be interpreted the other way round: the lack of forebrain MR resulted in working memory deficits in the water maze task because a functional GR facilitated the consolidation of nonadaptive behavior. We conclude that the observed behavior of animals with differential MR and GR conditions will only be understood in relation to the contribution of both receptors.

### 4.2. For optimal cognitive performance, not only MR but also moderate GR activation is necessary

ALC mice with MR and moderate GR activations display low anxiety during the first days of testing, general low arousal, and fast learning. Corticosterone levels in the ALC mice were continuously elevated in the range of the circadian rise, thus it would not be expected to cause damage to neurons, downregulation of MR and GR, or alterations in neurotransmitters implied in cognitive impairments [35]. In fact, ALC mice with MR and moderate GR activations showed the best cognitive performance.

Part of this improved learning and memory ability could be explained by the emotional state of the mice. Like ADX mice, ALC mice have low anxiety (and arousal) during the first days of learning which is correlated with increased motivation and good learning. Supporting our argument is the most recent finding of Herrero, that rats with low anxiety showed faster spatial learning together with increased hippocampal MR; opposite results were found in high-anxiety rats [36]. Stronger MR availability and activation might underlie the fast onset of learning, while GR are responsible for the consolidation of this context-related information. [7, 17, 37, 38]. Therefore, it is not surprising that ALC mice with a moderately activated GR display improved or normal cognitive performance compared to ADX mice with little or no GR activation throughout testing. For optimal coordination of cognition and emotion, both MR and a moderate activation of GR are necessary [39, 40].

### 4.3. Substantial continuous GR activation in addition to MR activation are associated with high emotional arousal and impaired learning

As described by many others, chronic strong GR activation caused by, for example, severe stressors or pharmacological modulation of the HPA axis results in impaired learning and memory [41–43], reduced synaptic plasticity in the hippocampus [44], increased anxiety [45], and even depression-like symptomatology [38]. In patients suffering form depression or Cushing's disease, elevated levels of cortisol have been associated with poorer cognitive performance in verbal memory, working memory, and post-encoding tasks [46–48]. Furthermore, an association between cortisol level and increased fear perception has been found in patients suffering from recurring depression [49], which also indicates a modulatory role of glucocorticoids in emotional processes.

We find similar results for emotions and cognition: AHC mice with MR and continuous high GR activation have a slow onset of learning together with increased arousal and anxiety-like behaviors and suppression of directed exploration. It is not surprising that these mice display a slower onset of learning (opposite to low anxiety and fast learning as described above). At first glance, it seems surprising that when learning starts to occur, the magnitude of learning (Figure 2(c): time to finish task, slope of the learning curve) is the same in ALC and AHC mice. The change in corticosterone availability, due to the encapsulation of the pellet, is most likely responsible for the altered behavior. Corticosterone levels decreased over the days to concentrations in the “normal” range, that is, comparable to circadian peak secretion and the amount of corticosterone measured in ALC mice at the beginning of testing. Thus, in AHC mice we deal with memory impairments and high emotional arousal only during specific stages of learning, namely during the first days of testing that coincide with really high exposure to corticosterone.

### 4.4. The highly anxious sham-operated control group

**Figure 3:**
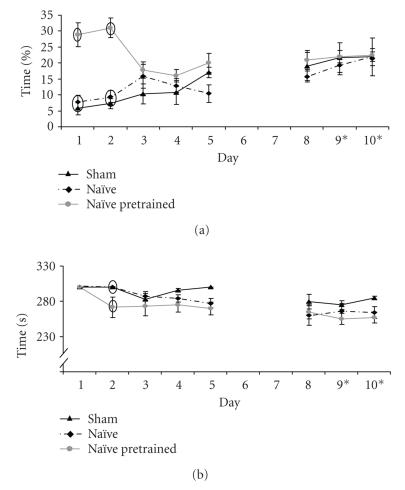
Examples of behavior of the mice during the followup experiment. (A) Percentage of time spent on center board. (B) Time to finish the task (5 minutes or finding all three baits) of sham (black line), naïve (striped black line), and naïve mice preexposed to a bait-containing cylinder in the homecage (grey line). Days 9 and 10 on the *x*-axis indicate removal of rings from all cylinders, while the bait remained in the same cylinders. Data present the mean of the three trials per day ± SEM. Ovals mark data points with significant differences: *P* < .05 between groups within days.

We used sham-operated mice that have an intact HPA axis as control group. Unexpectedly, these mice were characterized as highly anxious and with little motivation, with high arousal and a slow onset and little progress of learning. Factor 1 was significantly different over time between sham and all other groups tested: low motivation and high anxiety throughout testing days. We got the impression that the behavioral setting remained anxiogenic to these mice. Lack of exploration of the centre board might also prevent learning basic rules, for example, that cylinders are baited with almonds. This and the possible role of a prolonged effect of surgery on the HPA system resulted in a follow-up experiment. We used three groups of mice (*n* = 6 per group): (1) sham-operated mice and (2) naïve, nonoperated mice received almonds in the homecage to familiarize with the bait, like the experimental groups, (3) naïve mice received almonds in the cylinders four times in the week before the modified hole board task. Sham and naïve mice without preexposure to the cylinders displayed similar high anxiety and slow learning as we saw before. However, after pretraining with baited cylinders anxiety decreased, motivation increased and learning improved (Figure 3).

Since surgery did not influence behavior on the modified hole board, incomplete recovery from the surgery is unlikely to affect performance. Using a somewhat different experimental design, comparably long times to finish the task have been reported for C57BL/6 mice (Ohl 2003; still 280 to 300 seconds after eight days of training). In contrast, prior familiarization to items of the test condition reduced anxiety-like behavior and increased motivation, which could (in part) increase cognitive performance like it was observed in ADX and ALC mice.

It is remarkable that mice without adrenals dysregulated HPA-axis activity and additional pellet implantation “did better” compared to the relative intact sham and naïve control groups. These findings even more underscore that (i) high anxiety and arousal have negative consequences for cognition while (ii) less anxiety, increased motivation, and goal-directed exploration have a positive influence on behavior (see also [36]). We consider the role of MR in the integration of sensory information and behavioral strategies central for reduced anxiety-related behavior.

### 4.5. Adrenalectomy: other hormones and anxiety

The adrenalectomy-induced deficit in corticosterone secretion results in the disinhibition of HPA activity, and thus enhanced release of corticotrophin-releasing hormone (CRH) and vasopressin (AVP) from the hypothalamus. Also the adrenal medulla as source of adrenaline is eliminated. CRH, AVP, and adrenaline, all might play a role in emotional expressions and cognitive performance [50] of ADX mice, with and without supplementary corticosterone.

Considering the function of the GR in the negative feedback, we may expect that ADX mice (predominant MR activation) and ALC mice (MR and moderate GR) have a deficient suppression of CRH and AVP activities [51, 52]. Mice with elevated levels of CRH that acts predominantly via CRH receptor 1 are expected to display increased anxiety. Mutant mice with a deficient CRH receptor 1 either by genetic deletion or pharmacological blockade are less anxious [53]. Clearly, CRH is involved in anxiety-related behavior. However in the present study, ADX and ALC mice show low anxiety-related behavior, while AHC mice (predominant GR activation) are highly anxious. These findings do not support a role of hypothalamus-related CRH activity in anxiety behavior in the present study. The same argument holds true for AVP.

In response to stress, noradrenalin release increases. This is thought to contribute to the anxiogenic effects of stress [50, 54], in which the amygdala plays an important role [55]. AHC and sham mice showed the strongest arousal (defecation) and were characterized as most anxious: a participation of catecholamines in these responses cannot be excluded. Furthermore, changes in metabolism and food intake have to be considered. Although food was present *ad libitum* throughout the experiment and body weight did not differ between the groups, motivation to go for the almond-bait might have been increased in ADX and ALC mice. Factor analysis also underlines the role of motivation in relation to anxiety for the performance.

### 4.6. Less directed exploration: is this anxiety?

Anxiety-related behavior in rodents is generally deduced from the avoidance of an open, bright, and unprotected area. However, tasks characteristics largely influence behavior. For example, rats that are specifically selected for their avoidance of open arms of the elevated plus maze, and thus classified as high anxiety rats, do not avoid the center (open) area of a hole board task [56]. Complexity and duration of the task, as well as motivational aspects might overcome state anxiety. Directed exploration or behavioral reactivity is expressed by approach to certain stimuli, for example, the number of visits to a specific location in the testing area. These opposing behaviors are both related to locomotor activity. Does directed exploration rely on reduced anxiety? In the present study, animals with low directed exploration would spend little time near the cylinders on the centre board. The interpretation of this behavior could be high anxiety. Although it is likely that anxiety interacts with directed exploration, this does not necessarily has to be the case. It could be that our interpretation of high anxiety is characteristic for a more passive exploration strategy [57, 58] without a dominant role for anxiety-related behavior. The setting of our task and subsequent factorial analysis allowed us to differentiate anxiety-like behavior from directed exploration: they did not coincide into one factor, indicating no correlation between the two.

## 5. CONCLUSION

Anxiety and motivation are important factors for the onset of learning, a process in which MR and GR and their coordinated activation play a crucial role. Continuous predominant MR activation appears to be beneficial for the emotional state, resulting in low anxiety, high motivation, and high directed exploration and behavioral reactivity, but does not result in better learning and memory. Additional moderate GR activation also results in low anxiety and high motivation, with the advantage of improved cognition expressed as a decrease in working memory errors. In contrast, MR with additional substantial GR activation results in a slow onset of learning together with high anxiety, showing similarities with patients suffering from depression and Cushing's disease. We conclude that optimal performance is bound to continuous MR activation together with moderate GR activation. Further increase in corticosterone, and therefore substantial GR activation, will increase emotional arousal with impairing effects for learning and memory.

## Figures and Tables

**Table 1:** Behavioral parameters measured in the modified hole board.

Total number	Sit

—	Rearing
—	Stretched attend
—	Grooming
—	Center board entries
—	Hole visits
—	Baited holes visited
—	Nonbaited holes visited
—	Repeated hole visits
—	Baits obtained
Latency	First center board entry
—	First hole visit
—	Eat bait
Time	Sit
—	Grooming
—	On center board
—	To finish task

**Table 2:** Principal component analysis over all data, with varimax rotation and Kaiser normalization. Behavioral parameters are represented as factor loading per factor. Factor loadings with equal value are positively correlated, while loadings with opposing values are negatively correlated. Loadings *<* 0.6 are not included in this table. Eleven of the seventeen measured parameters (Table 1) have communalities *>* 0.7 and are included in the factor analysis.

	Factor			
	
	1	2	3	4
	
	Anxiety, motivation, good learning	Directed exploration/behavioral reactivity, working memory	General activity	Impaired learning

Latency to eat bait	−0.887	—	—	—
Number of baits obtained	0.862	—	—	—
Latency to first hole visit	−0.792	—	—	—
Number of baited holes visited	0.781	—	—	—
Time on center board	0.678	—	—	—
Number of repeated hole visits	—	0.927	—	—
Number of hole visits	—	0.807	—	—
Time sitting	—	—	0.840	—
Number of rearings	—	—	−0.810	—
Number of nonbaited holes visited	—	—	—	0.911
Ratio of right hole visit/ % and wrong hole visits %	—	—	—	−0.723

**Table 3:** Plasma corticosterone, thymus, and body weight. Corticosterone was measured before the first day of testing (day 0) and 24 hours after the last testing day (day 11). Data are presented as mean ± SEM.

	Plasma corticosterone (ng/mL)		Thymus weight (mg)	Body weight (g)

Group	Day 0	Day 11	Day 11	Day 11

Sham	13.78 ± 2.37	17.96 ± 4.10	49.3 ± 0.9	25.1 ± 0.8
ADX	12.39 ± 1.50	15.24 ± 8.81	64.2 ± 2.5*	27.4 ± 0.7
ALC	88.67 ± 19.26*	33.18 ± 4.87	38.9 ± 0.5*	24.7 ± 0.7
AHC	168.00 ± 19.23*	88.63 ± 10.58*	21.2 ± 1.2*	25.3 ± 1.2

*P < .05 compared to all other groups.
